# Radiotherapy intensification for atypical and malignant meningiomas: A systematic review

**DOI:** 10.1093/nop/npad077

**Published:** 2023-12-18

**Authors:** Simona Gaito, Love Goyal, Romelie Rieu, Anna France, Neil G Burnet, Claire Barker, Shermaine Pan, Rovel J Colaco, Giuseppe Minniti, Federico Roncaroli, Ed Smith, Marianne Aznar, Gillian Whitfield

**Affiliations:** Proton Clinical Outcomes Unit, Christie NHS Proton Beam Therapy Centre, Manchester, UK; Division of Cancer Sciences, School of Medical Sciences, Faculty of Biology, Medicine and Health, University of Manchester, Manchester, UK; Department of Proton Beam Therapy, Christie Proton Beam Therapy Centre, Manchester, UK; Department of Proton Beam Therapy, Christie Proton Beam Therapy Centre, Manchester, UK; Institute of Cancer Research, London, UK; Head and Neck Unit, Royal Marsden Hospital, London, UK; Proton Clinical Outcomes Unit, Christie NHS Proton Beam Therapy Centre, Manchester, UK; Department of Proton Beam Therapy, Christie Proton Beam Therapy Centre, Manchester, UK; Department of Proton Beam Therapy, Christie Proton Beam Therapy Centre, Manchester, UK; Department of Proton Beam Therapy, Christie Proton Beam Therapy Centre, Manchester, UK; Department of Proton Beam Therapy, Christie Proton Beam Therapy Centre, Manchester, UK; Department of Radiological Science, Oncology and Anatomical Pathology, Umberto I Hospital, University Sapienza, Policlinico Umberto I, Rome, Italy; IRCCS Neuromed, Pozzilli, Italy; Division of Neuroscience, Geoffrey Jefferson Brain Research Centre, School of Biological Sciences, Faculty of Biology, Medicine and Health, University of Manchester, Manchester, UK; Proton Clinical Outcomes Unit, Christie NHS Proton Beam Therapy Centre, Manchester, UK; Division of Cancer Sciences, School of Medical Sciences, Faculty of Biology, Medicine and Health, University of Manchester, Manchester, UK; Department of Proton Beam Therapy, Christie Proton Beam Therapy Centre, Manchester, UK; Division of Cancer Sciences, School of Medical Sciences, Faculty of Biology, Medicine and Health, University of Manchester, Manchester, UK; Division of Cancer Sciences, School of Medical Sciences, Faculty of Biology, Medicine and Health, University of Manchester, Manchester, UK; Department of Proton Beam Therapy, Christie Proton Beam Therapy Centre, Manchester, UK

**Keywords:** malignant meningiomas, proton therapy, radiotherapy intensification

## Abstract

**Background:**

The outcomes of nonbenign (WHO Grades 2 and 3 [G2, G3]) meningiomas are suboptimal and radiotherapy (RT) dose intensification strategies have been investigated. The purpose of this review is to report on clinical practice and outcomes with particular attention to RT doses and techniques.

**Methods:**

The PICO criteria (Population, Intervention, Comparison, and Outcomes) were used to frame the research question, directed at outlining the clinical outcomes in patients with G2−3 meningiomas treated with RT. The same search strategy was run in Embase and MEDLINE and, after deduplication, returned 1 807 records. These were manually screened for relevance and 25 were included.

**Results:**

Tumor outcomes and toxicities are not uniformly reported in the selected studies since different endpoints and time points have been used by different authors. Many risk factors for worse outcomes are described, the most common being suboptimal RT. This includes no or delayed RT, low doses, and older techniques. A positive association between RT dose and progression-free survival (PFS) has been highlighted by analyzing the studies in this review (10/25) that report the same endpoint (5y-PFS).

**Conclusions:**

This literature review has shown that standard practice RT leads to suboptimal tumor control rates in G2–3 meningiomas, with a significant proportion of disease recurring after a relatively short follow-up. Randomized controlled trials are needed in this setting to define the optimal RT approach. Given the increasing data to suggest a benefit of higher RT doses for high-risk meningiomas, novel RT technologies with highly conformal dose distributions are preferential to achieve optimal target coverage and organs at risk sparing.

Meningiomas account for more than one-third of all primary intracranial tumors. According to the SEER database, their estimated incidence in the United States is around 6.3 per 100 000 individuals per year.^1^ Meningiomas are more common in women and are most frequently diagnosed between 40 and 70 years with an age peak at around 65 years.^2^ Mutational status correlates with anatomical location with 22q deletion and/or neurofibromatosis type 2 (*NF2*) mutations being common in convexity meningiomas and spinal meningiomas and mutations in *AKT1*, *TRAF7*, *SMO*, and/or *PIK3CA* genes typically occurring in skull base meningiomas.^3^ Chromosome gains are common, particularly in Grades 2 and 3 lesions.^4^

Meningiomas can also occur in genetic syndromes with NF2 being the most common. Others include Gorlin syndrome, *NF1*, *VHL*, and syndromes associated with mutations in *SMARCE1*, *BAP1*, *SUFU*, *PTEN*, and *CREBBP* genes. These syndromic meningiomas are often radiosensitive.

The fifth edition World Health Organization (WHO) classification of Central Nervous System tumors (CNS; CNS5 hereafter) stratifies meningiomas in benign (WHO Grade 1 [G1]), atypical (WHO Grade 2 [G2]) and anaplastic (or malignant, WHO Grade 3 [G3]) based on the historical pathological criteria including mitotic count and architectural and cytological features, histotype and molecular profile [G3]. In G1 meningiomas, the Ki-67 labeling index higher than 4% and higher than 20% has been found to correlate with higher recurrence rates respectively similar to G2 and G3 meningiomas.^5^ Unlike previous classifications, mutations in the TERT promoter and homozygous loss of CDKN2A define a WHO (G3) meningioma, irrespective of histotype. Clear cell and chordoid meningioma are still defined as G2 and rhabdoid and papillary as G3 although the histotype itself is not enough to define their grade. The same criteria for atypical should be present to define them as G2 or G3. Genetic alterations are also relevant. For instance, rhabdoid meningiomas behave aggressively when they bear *BAP1* gene mutations.^5^ Invasion of the adjacent gray matter is still regarded as unfavorable feature in CNS5 although this view is not uniformly accepted.^6,7^ The classification of meningiomas has been further implemented with the introduction of genome-wide DNA methylation arrays.^8^ A recent score that combines microscopic features, gene copy number variation, and epigenetic profile proved more accurate in the prognostic stratification of meningiomas.^9^

The treatment of aggressive meningiomas includes a combination of surgery, radiation therapy (RT), and, in some cases, systemic treatment. The treatment approach depends on several factors including tumor size, location, extent of resection and histotype, WHO grading, molecular profile as well as patients’ overall performance status. The optimal management should be discussed and agreed upon in multidisciplinary settings including neurosurgeons, neuroradiologists, neuropathologists, radiation oncologists, and medical oncologists.^10–12^

The use and timing of adjuvant radiotherapy (ART) in atypical meningiomas (G2) after gross total resection (GTR) is still debated and it is currently being investigated in the ROAM trial.^13^ The upfront use of ART is less controversial in “high-risk” meningiomas, as defined in the RTOG 0539 trial.^14^ This group includes G2 and G3 meningiomas with macroscopic residual disease after surgery/biopsy as well as unresectable and recurrent disease. The general consensus is to treat this group of patients with 60Gy to the high-risk volume (residuum) and 54Gy to the low-risk,^15^ but practice varies among institutions.^14^

The RTOG 0539 trial reported suboptimal control rates of high-risk meningiomas, with 3-year progression-free survival (PFS) of 50%–60% that drops when patients are followed for longer periods.^14^ Dose intensification strategies have been tested in clinical trials or documented in retrospective series.^16^ However, due to the proximity to critical structures, the optimal management needs to consider that tumor-related morbidity and treatment-related toxicities can be considerable. For this reason, efforts should be directed toward improving tumor control rates without jeopardizing treatment tolerance.

Current radiotherapy modalities such as Intensity Modulated Radiotherapy (IMRT), Volumetric Modulated Arc Therapy, and particle therapy can deliver high-dose very precisely to the tumor area.^17–19^ Proton beam therapy (PBT) can deliver higher doses without increasing the risk of side effects due to the lack of exit dose beyond the target.^20,21^ Reduction of low-dose bath or on occasion its elimination leads to a reduction of acute and late toxicities. For these physical characteristics, PBT is being investigated as a treatment option for dose intensification strategies.

The aim of this review is to appraise the clinical practice of RT doses and treatment outcomes in high-risk meningioma patients.

## Methods

To facilitate the identification of relevant information, the research question was framed following the PICO criteria (Population, Intervention, Comparison, and Outcomes)^22^ as reported subsequently.

Patients with anaplastic or atypical or G2 or G3 or high-risk meningioma represented the “*population*” and radiotherapy or radiation therapy or proton therapy or PBT or particle therapy were the “*intervention*.” There was no specific “*comparison”* for this study. The “*outcomes”* focused on clinical outcomes OR efficacy.

Search strategy: (exp meningioma/ or “malignant meningioma”/ or (meningioma or ((meninges or meningeal) adj2 (tumo?r or tumo?rs))).ti,ab.) and (exp Treatment Outcome/ or disease-free survival/ or progression-free survival/ or event free survival/ or (((treatment or clinical) adj (effective* or efficacy or outcome*)) or rehabilitation or ((disease or local) adj control) or ((event or progression or disease) adj free survival)).ti,ab.) and ((“proton* therap*” or “proton beam therap*” or protontherap* or “proton beam radiation therap*” or “proton adiotherapy*” or pbt or “particle therapy” or radiotherapy or “radiation therapy” or “Photon Beam Therap*” or “Photon Therap*” or “Intensity modulated radiotherapy” or “IMRT” or (“adjuvant” adj2 (“radiation” or “Radiotherapy”))).ti,ab. Or Proton Therapy/ or Radiotherapy/).

**Figure 1.**
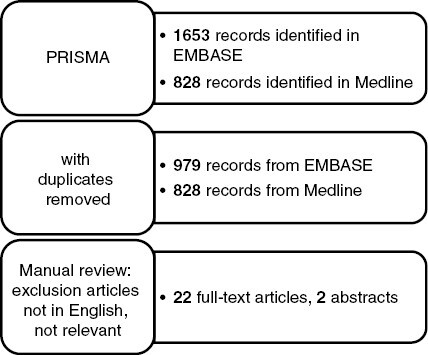
A preferred reporting items for systematic reviews and meta-analysis (PRISMA) flowchart used for selecting the relevant articles for this systematic review.

As of July 2023, this search strategy returned 2 481 entries in total between Embase (1 653) and MEDLINE (828). After deduplication, 1 807 records remained (979) from Embase and 828 from MEDLINE. This list of articles was split between the coauthors of this manuscript for manual review. Exclusion criteria included: articles not in English, off-topic, retrospective series treated with extreme hypofractionation or radiosurgery, total prescription doses <54 Gy/GyRBE, and literature reviews. After this selection process, 25 records (23 full-text articles and 2 abstracts) were included in this review (Figure 1).

## Results

The relevant clinical features and treatment modalities described in the studies included in this review are shown in Tables 1 and 2. Articles have been grouped into those including pure G2 meningiomas and those including G2–G3 or G1–G2–G3 meningiomas. Results from G2–G3 meningiomas have been extrapolated from the cohorts of mixed G1–G3 cases.

**Table 1.** Details of the Studies Presented in the review.

No.	Author	Article type	Year	Site	Age (median, range)	No. of patients	Grade	Type of study	Particle therapy (Y/N) and proportion	Follow-up (median, range)	New vs Recurrence^*^	Reirradiation (Y/N) and proportion	Other specifics
1	Press^23^	F	2013	Brain, BoS	53 (21−88)	46	2	Retrospective	N	26 m (7−107)	N/S	N	
2	McDonald^24^	F	2015	Brain, BoS	42 (14−75)	22	2	Retrospective	Y, 100%	39 m (7−104)	N + R	N	RI 6/ 22^†^
3	Weber^16^	F	2018	Brain, BoS	54 (28−72)	56	2	Phase II trial	N	5.1 y	N	N	
4	Hemmati^25^	F	2019	Brain, BoS	59 (22−84)	99 (19 ART)	2	Retrospective	N	37 m	N	N	
5	Lee^26^	F	2020	Brain, BoS	55.3 (45.7−66.5)	230 (51 ART)	2	Retrospective	N	6.9 y (1.4−18.8)	N/S	Y (4%)	NF2 9/230^‡^
6	Bender^27^	F	2021	Brain, BoS	58(19−88)	45	2	Retrospective	N	6.7 y	N + R (31%)	N	
7	Hoffmann^28^	F	2021	Brain	64 (*SD* 13.2)	31	2	Retrospective	N	4.39 y (0.13−10.45)	N + R	N	
8	Deng^29^	F	2022	Brain, BoS	49.5 (15−75)	44 (33 high risk)	2	Retrospective	Y, 43%	78 m (28−258)	N	N	
9	Byun^30^	F	2022	Brain, BoS	N/S	518 (158 ART)	2	Retrospective	N	64.9 m	N	N	
10	Hug^31^	F	2000	Brain, BoS	49(6−79)	31	2−3	Retrospective	Y, mixed PBT/XRT	59 m (7−155)	N + R (41%)	Y (10%)	RI 3/31^†^
11	Pasquier^32^	A	2008	Brain	57.6 ± 12	119	2−3	Retrospective	N	4.1 y	N + R (21%)	Y (4%)	
12	Boskos^33^	F	2009	Brain, BoS	52.5(20−72)	24	2−3	Retrospective	Y, mixed PBT/XRT	45.5 m (11−87)	N + R (67%)	N	
13	Chan^34^	F	2012	Brain, BoS	46 (26−56)	6	2−3	Phase I/II trial	Y, mixed PBT/XRT	145 m	N	N	
14	Detti^35^	F	2013	Brain, BoS	58.5 ± 14	66	2−3	Retrospective	N	80.4 m (18−238.8)	N + R	N	
15	Zollner^36^	F	2018	Brain	59.7(26−79)	20	2−3	Retrospective	N	31 m (20.1−42)	N + R	N	
16	Roger^14^	F	2020	N/S	≥18	51	2−3	Phase II trial	N	48 m (2.4−75.6)	N + R (23%)	N	
17	Holub^37^	A	2021	Brain	61.7(22−79.8)	104	2−3	Retrospective	Yes, 100%	39.4 m (1.2−225.8)	N	N	
18	Kent^38^	F	2021	Brain	60 (49−69.5)	66(28 ART)	2−3	Retrospective	N	8.2 y (7.4−13.1)	N	N	
19	Goldsmith^39^	F	1994	Brain, BoS	49 median	140 (23 HG)	1−3	Retrospective	N	40 m	N + R	N	
20	Noel^40^	F	2002	Brain, BoS	47 (11−71)	17	1−3	Retrospective	Y, mixed PBT/XRT	37 m (17−60)	N + R (70%)	N	
21	Milker−Zabel^41^	F	2007	Brain, BoS	57.2(13.3−79.2)	94 (13% HG)	1−3	Retrospective	N	52.8 m (1.6−82.7)	N + R	Y (0.01%)	
22	Weber^42^	F	2012	Brain, BoS	48.3 ± 17.9	39	1−3	Retrospective	Y, 100%	54.8 m (6.2−146.8)	N + R (18%)	Y (15.4%)	
23	Combs^43^	F	2012	Brain, BoS	53 (16−83)	507 (35 HG)	1−3	Retrospective	N	107 m (1−270)	N + R (25.8%)	N	
24	Kaul^44^	F	2014	Brain, BoS	59 (20−27)	297 (32 HG)	1−3	Retrospective	N	35 m (1−132)	N	N	
25	Murray^45^	F	2017	Brain, BoS	52.8 (3−77)	96	1−3	Retrospective	Y, 100%	56.9 m (12.1−207.2)	N + R (44.8%)	N	

*Notes*: A= abstract; ART = adjuvant radiotherapy; BoS = Base of Skull; F= full article; m = months; y = years; GTR = Gross total resection; HG = high grade (II + III); N = New; N = No; N/S = not specified; RT = radiotherapy; pts = patients; PBT = proton beam therapy; R = Recurrence; XRT = X-Ray radiotherapy; RI = radiation-induced; NF 2 = Neurofibromatosis; Y = yes.

^*^When specified in the study, the percentage of recurrent disease is reported in brackets.

^†^These studies report the number of presumably radiation-induced diseases.

^‡^This study reports the number of patients with Neurofibromatosis 2 in this cohort.

**Table 2.** Treatment-Related Characteristics of the Studies Presented in the Review.

No.	Author	No. of patients	Total dose (Gy or GyRBE)	No. of fractions	Dose per fraction (Gy or GyRBE)	Local control/ progression free survival rates	Acute and late toxicities	Risk factors for worse outcomes
1	Press^23^	46	59.4 (49.2−61.2)	33(27−34)	1.8	2-y LC 92%; 3-y LC 74%	1 G3 CNS necrosis	Distinct pathologic features
2	McDonald^24^	22	63(54−68.4)	30−38	1.8	5- LC- 71.1%	1 G3 CNS necrosis	RT dose ≤ 60Gy
3	Weber^16^	56	60	30	2	3y PFS: 88.7%; 3y OS: 98.2%	G ≥ 3 LT 14.3%	N/S
4	Hemmati^25^	99 (19 ART)	59.4 (54−59.4)	30−33	1.8	median PFS 64 m (ART group)	N/S	Surgical extent, no RT
5	Lee^26^	230 (51 ART)	59.4 (13−59.4)	33 (1−33)	1.8 (1.8−15)	5y and 10y PFS after GTR + ART: 94%; 5y and 10y PFS after STR + ART: 69% and 43%	N/S	delayed RT
6	Bender^27^	45	60	30	2	5y PFS:53%	N/S	Ki67, extent of resection, delayed RT
7	Hoffmann^28^	31	54.0−59.4/60	30−33	1.8−2.0	5 y PFS 67.9% (SE 10.5%)	No G ≥ 3 AT and LT	Mib-1 above the median
8	Deng^29^	44 (33 high risk)	60 (54−68)	N/S	1.8−3	3y PFS 86.2%	G ≥ 3 LT: 6/44 (13.6%)	DNA methylation
9	Byun^30^	518 (158 ART)	60	30	2	5y PFS in ART group: 80.8% (71.9% after STR)	N/S	Surgical extent, tumor size, Ki-67 index
10	Hug^31^	31	G2: 62.5 (50.4−68.4); G3 58 (40.4−72)	N/S	1.8−2	G2: LC 38% at 5y and 19% at 8y; G3: LC 52% at 5y and 17% at 8y	LT:1 G3, 2 G4	Dose ≥ 60Gy/GyRBE, combined PBT/XRT
11	Pasquier^32^	119	54.6 ± 5.1	N/S	1.8−2	5y DFS 58%; 10-y DFS 48%. 5y OS 65%; 10y OS 41%.	N/S	KPS, age, high mitotic rate
12	Boskos^33^	24	65−68	N/S	1.8−2	G2−G3: 3y LC: 61.3 ± 11; 5y LC: 46.7 ± 12.3	1 G3 LT	NO significant impact of any analyzed factor on LC
13	Chan^34^	6	68.4−72	38−40	1.8	LC at median f-up: 100% for G2, 50% for G3	No G ≥ 3 AT or LT	N/S
14	Detti^35^	66	54.6 (50−60)	25−30	2	5 y DFS: 76.5%	No G ≥ 3 AT and LT	Older age, RT dose (< 52 Gy), WHO grade, size > 5 cm
15	Zollner^36^	20	60 (59.4−60)	30−33	1.8−2	2 y PFS 87.5%; 3 y PFS 70%	No G ≥ 3 AT and LT	N/S
16	Roger^14^	51	60	30	2	3 y PFS -58.8%; 3 y LC 68.9%	LT: 16 G3;1 G5	
17	Holub^37^	104	68.4	38	1.8	5y PFS: 78.8%	G1−3 late tox:52.9%	XRT
18	Kent^38^	66(28 ART)	55.8(IQR 54−59.4)	31 (30−33)	1.8	in ART group: 5-y PFS 57.7%; 10-y PFS 37.7%	N/S	Ki67; delayed RT
19	Goldsmith^39^	140 (23 HG)	54(44.62−69.26)	30	2	5-y PFS 48% for G2–G3	G ≥ 3 LT in 5 pts (3.6%)	Older age, older RT techniques
20	Noel^40^	17	61(25−69)	N/S	1.8−2Gy	LC at 4y: 87.5 ± 12%^*^	No G ≥ 3 toxicity	N/S
21	Milker−Zabel^41^	94 (13% HG)	57.6 (50.4−62)	32	1.8	LC at 4.4 y: 93.6%^*^	1 G4 LT (reirr)	N/S
22	Weber^42^	39	60.8 ± 5.3	33−37	1.8	G 2−3, 5-y LC 49.1%	5 G ≥ 3 LT (31.3%)	High grade, tumor volume, male gender
23	Combs^43^	507 (35 HG)	57.6 (25−68)	N/S	1.8Gy (1.6−5)	G2−G3: 5-y LC 81%, 10-y LC 53%	N/S	Male gender
24	Kaul^44^	297 (32 HG)	nFSRT: 57.31 ± 5.82 hFSRT: 37.6 ± 4.4 SRS: 17.31 ± 2.58	N/S	1.6−2.2 Gy-nFSRRT2.2-5Gy- hFSRT	G2−G3: 3y PFS 52.6%, 5y PFS 19.7%	No G ≥ 3 toxicity	Hgh grade, no surgery
25	Murray^45^	96	62(54−68)	30−38	1.8	G2−3, 5-y LC 68%	Acute:1 Gr 3 (brain edema) Late:6 G3 (brain oedema, optic toxicities),2G4 (optic toxicities)	High grade, non-BoS location, male gender, recurrence

*Notes*: AT = acute toxicities; ATR = adjuvant radiotherapy; CNS = Central Nervous System; DFS = disease free survival; G = Grade; Gy = gray; GTR = Gross Total resection; LC = local control; LT = late toxicities; N/S = not specified; OS = overall survival; RT = radiotherapy; XRT = X-Ray radiotherapy; tox = toxicity; STR = subtotal resection; PFS = progression-free survival; RBE = relative biological effectiveness; SE = standard error; nFSRT = conventionally fractionated stereotactic radiotherapy; hFSRT = hypofractionated stereotactic radiotherapy; SRS = radiosurgery; y = year.

^*^Reported on the whole cohort.

Outcomes and toxicities were not uniformly reported in the selected studies. Different endpoints and time points were used preventing an accurate comparison across studies. Moreover, the toxicity for each tumor grade was not always specified.

### Studies on WHO Grade 2 Meningioma

#### Impact of Margin Reduction.—

Press et al.^23^ evaluated the impact of target volume margins on outcomes in 46 G2 meningiomas treated with IMRT including primary and recurrent lesions and 51% sub-totally resected tumors (STR). They concluded that margin expansions of ≤1 cm were not associated with increased recurrence rate at the margin as compared to other retrospective series. with 2- and 3-y Local Control (LC) rates of 92% and 74%, respectively, after ART to a median dose of 59.4 Gy (49.2–61.2). In this cohort, the cumulative number of pathologic risk factors (brain invasion, necrosis, equal or more than 4 mitoses/10 high power fields, nuclear atypia, clear cell or chordoid histology) seemed to worsen LC.

#### Molecular/Proliferation Markers and Patterns of Failure.—

Using a classification integrating microscopic features, copy number variation, and methylome classification, Deng et al. stratified 44 patients with G2 meningiomas into 3 risk groups (low, intermediate, and high-risk).^29^ Such risk stratification better-reflected differences in 3-year local progression-free survival (lPFS) following RT. In fact, the 3-year lPFS rates reported in this study ranged from 100% for the low-risk group to 75.5% for the high-risk group. The authors concluded that therapeutic management of G2 meningiomas and future clinical trials should integrate the molecular risk-groups, and not rely on microscopic grading alone.

Another parameter to identify patients at higher risk of local failure within G2 meningiomas was described by Hoffman et al.^28^ in a retrospective analysis of recurrence patterns and clinical outcomes of 31 patients after ART. They showed a higher tendency for re-growth in those with Ki67 above the median. Moreover, relapses after a median follow-up of 4.39 years almost invariably occurred in the high-dose region.

#### Radicality of Resection and Adjuvant Radiotherapy.—

Merging retrospective data from 4 different hospitals, Byun et al.^30^ proposed a risk stratification model based on 3 factors: tumor size, surgical extent, and Ki-67 labeling index. ART significantly improves PFS in patients with G2 meningiomas, irrespective of the risk category. The 5-year PFS rate for the subgroup undergoing ART was 80.8% versus 57.7% for the subgroup undergoing postoperative surveillance. The authors concluded that a prognostic model may guide decision-making for the use of ART. These results were corroborated by Lee et al.,^26^ who analyzed retrospective data from 230 patients specifically focusing on the timing of ART for G2 meningiomas. Upfront ART significantly improves PFS rates compared to delayed ART (*P* = .03), both after GTR and STR. The benefit of early ART became more evident after longer follow-up (>5 years).^26^ A trend of improved PFS and OS after ART that did not reach statistical significance was reported by Bender et al.^27^ in a retrospective cohort of 216 patients. Moreover, high Ki-67 and STR were significantly associated with a higher risk of recurrence (*P* < .05). Hemmati et al.^25^ reported in 99 patients with G2 meningiomas, 19 of whom treated with ART (median dose 59.4 Gy, range 54–59.4) and 80 without further postoperative treatment and observed PFS benefit in administering ART (64 m vs 37 m in the control group, *P* = .009). In multivariable analysis, a significant role of Simpson resection status was also shown (*P* = .032).

#### Impact of Dose Escalation.—

McDonald^24^ and co-authors reported the outcomes of 22 patients with G2 meningiomas treated with PBT between 2005 and 2013. The 5-year estimate of local control was 87.5% following a radiation dose >60 Gy (RBE), compared to 50.0% for ≤60 Gy (RBE; *P* = .038).

#### Reported Toxicities.—

Five of the 9 studies reviewed specifically report the incidence and severity of acute and late toxicities (AT and LT).^16,23,28,29,24^ The incidence of G ≥ 3 adverse events after ART ranged between 0% and 14.3%, mostly consisting of symptomatic radiation necrosis regressing after steroids or bevacizumab. Of note, Deng et al.^29^ report a higher incidence of G ≥ 3 LT following bimodal radiotherapy with a carbon ion boost of 18Gy RBE in 3Gy/ fraction after conventional photon radiotherapy of 50 Gy in 2 Gy/ fraction.

### Studies on Mixed Grades 2 and 3 Meningioma

#### Impact of Adjuvant Radiotherapy.—

The positive association between ART and improved PFS, regardless of extent of surgery was also described by Kent et al.^38^ in 66 patients with G2 and G3 tumors. Patients receiving ART were treated with fractionated RT to a median dose of 55.8 Gy (IQR, 54–59.4 Gy) in 1.8 Gy per fraction, with 5- and 10-year PFS rates significantly higher for the ART versus the surveillance group: 5-years PFS rates for ART versus surveillance were 57.7% versus 24.9%, and 10-y PFS rates for ART versus surveillance were 37.7% versus 12.4%, respectively. Kaul and colleagues^44^ reported the outcomes of RT in a mixed cohort of 297 patients treated between October 1995 and March 2005. Radiation therapy regimens included normo-fractionated, hypo-fractionated, and stereotactic radiotherapy. This study suggested that patients who received ART had better PFS as compared to patients treated with radiotherapy alone. There was no difference in outcomes between different fractionation schedules, however patients who had received conventionally fractionated RT showed a more favorable acute toxicity profile.

#### Advanced Radiotherapy Techniques and Dose Escalation.—

Goldsmith et al.^39^ were among the first authors to describe improved outcomes in patients with ART techniques to deliver higher radiation doses in G3 meningiomas with up to 69.2 Gy in conventional fractionation. They reported a 5-year PFS of 48% of patients. The positive association between precision X-ray radiotherapy (XRT) and improved LC was also described by Combs et al.^43^ in a retrospective series of 507 skull base meningiomas, 35 of which were G2–G3. LC rates for these lesions were suboptimal, being 81% at 5 years and dropping to 53% at 10 years. Holub et al.^37^ reported preliminary results of a dose escalation strategy to 68.4 GyRBE in 1.8GyRBE/ fraction in a cohort of 104 G2 and G3 meningiomas, with an encouraging PFS rate of 78.8% at 5 years. Detti et al. in 2013^37^ published the outcomes of 66 patients and reported that age and radiotherapy dose >52Gy were associated with a longer OS, while preoperative size and grading (G2 vs G3) influenced disease-free survival. Weber et al.,^42^ and subsequently.

Murray et al.^45^ on a larger cohort reported the long-term clinical outcomes of Pencil Beam Scanning (PBS) PBT for G1–G3 meningiomas. For the combined G2–G3 groups they reported 5-year LC rates of 49.1%^42^ and 68%.^45^ These outcomes were achieved through dose escalation up to 68 GyRBE and both described LC as being related to WHO grade and gender.

Noel et al.^40^ evaluated the efficacy and tolerance of escalated dose combining PBT/XRT in the treatment of benign, atypical, and anaplastic meningiomas and concluded that dose-escalation by 10%–15% may lead to clinical improvements in most patients as well as long-term stabilization, with encouraging LC rates for the whole G1–3 cohort (4-y LC 87.5 ± 12%).

Hug et al.^31^ reported the outcomes of combined radiotherapy with photons and protons in 31 patients with G2 and G3 meningioma. Doses for G2 tumors were 50–68Gy and 40–72Gy for G3 lesions. Analysis of LC following ART revealed that, for both G2 and G3 meningiomas, LC rates were significantly improved for prescribed target doses ≥60Gy, which were more often delivered with PBT.

#### Risk Stratification in Clinical Trials.—

The results of a phase II trial (RTOG 0539) assessing the safety and efficacy of risk-adaptive management strategies yielded 3-year PFS rates of 58.8% in the high-risk meningioma group for dose-escalated regimens, using a simultaneous integrated boost of 60 Gy to the high-risk volume in 30 fractions.^14^ In 2008, the European Organization for Research and Treatment of Cancer (EORTC) started a phase II study aimed at investigating the impact of high-dose RT on the PFS rate at 3 years in G2 and G3 meningiomas.^16^ All patients received ART. Those with Simpson G1–3 received 60 Gy in 30 fractions, whereas Simpson G4–5 received 70 Gy in 35 fractions, independently of the pathology grade. The analysis focused on the Simpson 1–3 group (combined G2–G3), which were the majority of patients, and 3-year PFS rates of 88.7% were reported for this subgroup.

#### Patterns of Failure.—

Zollner et al.,^36^ retrospectively investigated 20 G2–G3 meningiomas. They highlighted the risk of local recurrence in G3 lesions and the patterns of failure. After a median follow-up of 31 months (20.1–42), 4 recurrences were detected, 3 of which were in-field.

#### Reported Toxicities.—

Thirteen of the 16 papers investigated mixed cohorts specifically reported on the incidence of G ≥ 3 LT, which ranged between 0% and 32%,^14,39,31,33,35–37,40,42,44,45^ with the 11 G4^39,42,45,31,41^ and the 2 G5^14,39^ events described in total in the different series being almost invariably either brain radionecrosis or optic neuropathy.

## Discussion

**Figure 2.**
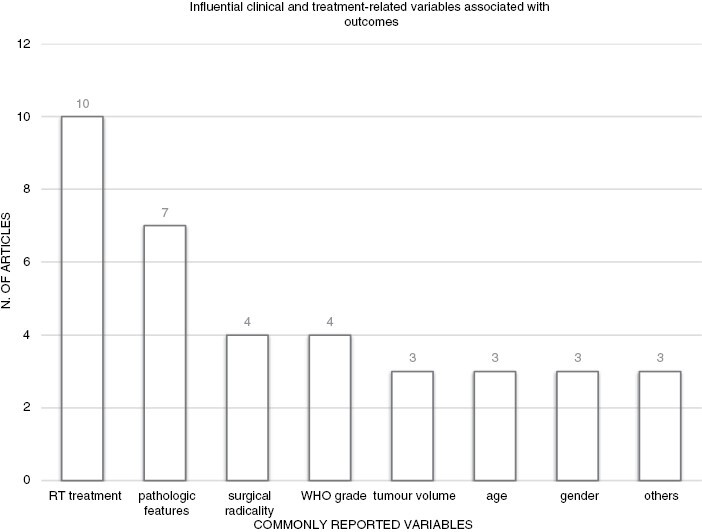
The most reported clinical- and treatment-related variables (grouped in bigger categories) described as associated with outcomes in atypical and malignant meningiomas in the studies presented in this literature review. RT treatment includes: Rt dose, technique and no/delayed RT.

The review and appraisal of outcomes of atypical and anaplastic meningiomas must consider the change in their classification from the third edition of WHO in 2000 and CNS5.^46,47^ Together with the WHO grading and the integrated microscopic and molecular profiling, other risk factors were highlighted in the articles presented in this review including large tumor size, subtotal resection, old age, delayed or no ART, lower radiotherapy doses (<52 Gy), or older RT techniques (Figure 2).^32^ Of note, RT showed the strongest correlation with prognosis. Advances in RT treatment deliveries and image guidance allowed for the safe delivery of higher doses to the targets without compromising treatment tolerance to unacceptable levels. Pathologic features include: mib-1/ki67, brain invasion, methylation status, etc. Others include: Performance status, location (base of skull vs non), and recurrent disease.

**Figure 3.**
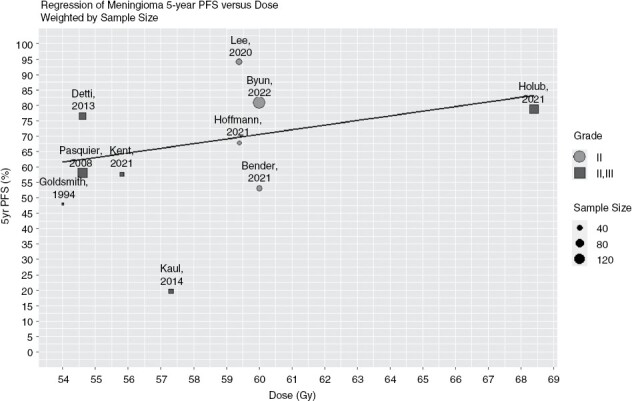
Regression of Meningioma 5-y PFS versus Dose (weighted by sample size). The circles refer to the pure G2 series, the squares to the mixed G2–G3 series. The size of the dots is proportional to the cohort size of each study. Of note, only the studies reporting the same endpoint (5-y PFS) have been included in this analysis.

A positive association between RT dose and progression-free survival (PFS) has been highlighted by analyzing the studies in this review (10/25) that report the same endpoint (5y-PFS), Figure 3.

The impact of ART in this patient population and its benefit on outcomes compared to post-operative surveillance is well described in studies investigating G2 meningiomas^25–27,30^ and in studies on combined G2–G3.^38,44^

The assumption that dose escalation can lead to improved outcomes in atypical and anaplastic meningiomas originated from a landmark study published in 1995, which demonstrated improved outcomes for patients receiving >50 Gy.^48^ These results have later been corroborated by several authors in studies on aggressive meningiomas.^28, 31,37,40,42,45,35^

In the risk stratification of meningiomas, the RTOG 0539 trial grouped “high-risk” lesions including G3 meningioma of any extent of resection, recurrent WHO G2 of any extent of resection extent, and new WHO G2 after subtotal resection.^14^ However, the reported PFS rates remained suboptimal, at 58.8% at 3 years despite the use of dose intensification to up to 60Gy.

The phase II EORTC 22042–26042 trial followed a different approach for risk stratification, stressing the importance of radical removal over WHO grading. The trial compared GTR versus STR regardless of grading and escalated to higher doses only STR lesion.^16^ The relevance of radical surgical removal has only been described retrospectively in G2 meningiomas^30,27,25^ and in fact the trial was powered to demonstrate a 3-year PFS > 70% in the G2 GTR group which had received 60 Gy in 30 fractions. Due to the very limited number of patients with G3 GTR and G2–G3 STR, the results of this trial were inconclusive for the other patient subgroups. In a comprehensive molecular analysis of the EORTC 22042–26042 trial, Maas et al. have shown the independent prognostic impact of DNA methylation class and chromosome 1p loss in WHO G2 and 3 meningiomas undergoing adjuvant high-dose radiotherapy. Multivariate analysis on the subset of patients with available molecular data shows that loss of chromosome 1p and methylation class were identified as significant risk factors for disease progression. However, due to the limited number of WHO G3 cases and cases with residual disease with adequate molecular information available, further analysis on these subgroups could not be performed and larger prospective cohorts are advocated.^49^

Three of the 7 studies reviewed in the review with a median RT prescription dose >60Gy used solely PBT and 4 used a combination strategy of PBT and XRT, with incidence of moderate-severe toxicities comparable to historical series using ≤60 Gy. McDonald et al.^24^ demonstrated that doses >60 Gy were associated with improved LC and a slightly reduced risk of intra-axial disease dissemination in atypical meningiomas.^24^ This data is consistent with the results from the small phase I/II dose escalation trial for high-grade meningiomas by Chan et al.^34^ suggesting promising long-term control with dose-escalated PBT. A large retrospective series from the same Institution (Hug et al., 2000^31^) further confirmed these findings. The main limitation of these studies was the small number of patients preventing from the identification of possible confounding factors and the impact on the outcome of variables such as different surgical procedures and radiation techniques. Moreover, the lack of randomized evidence precludes the estimation of the benefit that PBT may offer over XRT. In addition, all the studies analyzing the patterns of failure^23,28,36^ demonstrated that >80% of these occur in the high-dose region, suggesting that the use of smaller margins due to RT advances (neuroimaging, image registration for target delineation, radiation treatment delivery, and image guidance for patient localization) has not compromised tumor control outcomes and perhaps the use of current regimens is not sufficient to eradicate the disease.

Drawing definitive conclusions on radiotherapy schedules and recommendations on risk-adaption strategies from the studies presented in this review is not possible, due to their retrospective nature, and their limitations. In fact, the proportion of patients with a higher risk of disease recurrence such as recurrent diseases, subtotal resection, large tumor volume, and high proliferation differed in each series. Moreover, the reported clinical endpoints (PFS, LC, and OS) varied across studies, as well as the definition of time intervals for events censoring (ie, from surgery, from the end of radiotherapy, including the studies which report the same endpoint, such as those shown in Figure 3).

## Conclusions

Despite the limitations of retrospective studies, our review showed that standard practice RT leads to suboptimal tumor control rates in “high risk” WHO G2 and G3 meningiomas with a significant proportion of disease recurring after a short time. This is less so for “intermediate risk” disease, totally resected G2 tumors, where the role of adjuvant RT has been long debated and investigated in the ROAM trial.^13^ The integration of histological grading with genetic and epigenetic profiles provides a more accurate prognostic stratification, but it is not widely used in clinical practice. Integrated reports will help identify patients who may benefit from dose escalation. Robustly designed randomized controlled trials are needed to define the optimal radiation dose for G2 and G3 meningiomas. Given the increasing data to suggest a benefit of higher RT doses, PBT might be preferable to achieve optimal target coverage and OARs sparing. The increased availability and reduced costs of PBT could potentially include these conditions in the commissioned indications, which would ultimately widen the therapeutic window.
